# Risks of requiring a dedicated molecular specimen for HIV diagnosis and a potential strategy for mitigation

**DOI:** 10.1371/journal.pone.0237580

**Published:** 2020-08-13

**Authors:** Adam L. Bailey, Neil Anderson

**Affiliations:** Department of Pathology and Immunology, Washington University School of Medicine, St. Louis, MO, United States of America; Food and Drug Administration, UNITED STATES

## Abstract

**Background:**

HIV screening (*i*.*e*. antigen/antibody) tests are followed by a supplemental (*i*.*e*. antibody-only) if the screen is positive. Discrepant results can result from two scenarios: a false-positive screening test or acute HIV infection. These scenarios can be distinguished by a molecular HIV test, but due to contamination concerns, our laboratory recently implemented a policy requiring a second specimen dedicated for molecular HIV testing. Our objective was to (1) characterize the effect of this policy on the time-to-diagnosis for patients with discrepant screening and supplemental test results, and (2) explore “strength of positivity” as an interim predictor of screening test accuracy while awaiting confirmatory test results.

**Methods:**

Data from our laboratory information system, electronic health record, and instrument logs were used to collate data for all HIV testing performed at Barnes-Jewish Hospital (BJH) between January 1, 2014 and October 18, 2017.

**Results:**

Requiring a dedicated specimen for molecular testing significantly increased the time-to-diagnosis for patients with discrepant screening and supplemental HIV tests (p = 0.0084). This policy also contributed to loss-to-followup, with 0/35 discrepant cases lost-to-followup prior to policy implementation compared to 2/10 after implementation. However, by optimizing the signal-to-cutoff (S/CO) ratio of the screening test, we were able to more accurately distinguish false-positives from acute-HIV prior to molecular testing (sensitivity of 100%, specificity of 89%).

**Conclusions:**

We propose utilizing quantitative fourth-generation assay results (S/CO) ratios as a predictor of infection true positivity in situations where the screening assay is reactive but the supplemental test is negative and confirmatory molecular results are not immediately available.

## Introduction

The detection of HIV-specific antibodies in a patient’s serum has traditionally been required to make the diagnosis of HIV infection. However, third generation antibody-based assays are likely to miss cases of acute HIV infection, during which time viral loads are high but HIV-specific antibody titers have not yet risen [1, 2]. To address this shortcoming, the US Department of Health and Human Services recommends screening for HIV infection with an assay capable of detecting both HIV antibodies and HIV p24 antigen–a “fourth-generation” assay–as the first step in a sequential HIV testing algorithm. Reflexive testing of a reactive fourth-generation test with an antibody differentiation assay–a “supplemental” assay–is used to confirm HIV infection. Supplemental assays are second generation assays, meaning that they only detect HIV-specific IgG and are therefore unreliable in the acute phase of HIV infection. In cases where the fourth-generation screening test is positive but the supplemental test is negative, a molecular HIV testing is recommended to distinguish patients with acute HIV from those with a false-positive fourth-generation assay.

While not FDA cleared for this purpose, HIV viral load assays are more readily available than qualitative assays and are often used for confirmatory testing. These assays are highly-sensitive and are particularly susceptible to false-positives via sample contamination. As such, the College of American Pathologists (CAP) cautions against molecular testing on specimens that have been accessed in an environment where multiple specimens are accessed by an instrument without thorough decontamination between specimen samplings (CAP checklist item MOL.32360), as is done in most core laboratories where fourth-generation and supplemental HIV serology is performed. Indeed, carryover of viral RNA between specimens on automated lines–including the Abbott Architect immunoassay platform, which our laboratory uses for fourth-generation testing–has been documented [3, 4], and some false-positive HIV viral load tests are thought to be due to contamination of specimens during serologic testing.

Given these concerns, our laboratory recently instituted a policy whereby viral load testing was no longer performed on a specimen accessed outside of the molecular pathology laboratory; since August 1, 2016, we have required that a second specimen be obtained and dedicated for molecular testing. One year after implementation of this policy we performed a retrospective analysis to examine the impact of this policy on the time-to-diagnosis with respect to patients who relied upon HIV viral load testing for their diagnosis; *i*.*e*., patients who were reactive by fourth-generation assay but negative by supplementary HIV antibody differentiation assay. We further assessed the utility of using the signal-to-cutoff (S/CO) ratio generated by the fourth-generation assay as a predictive surrogate for discriminating between patients with acute HIV and patients with false-positive fourth-generation screening assays, which may be useful to triage clinical decision making in situations in which a second sample is not immediately available for molecular testing.

## Materials and methods

Records for all HIV fourth-generation, supplemental, and viral load tests performed at Barnes-Jewish Hospital (BJH) between January 1, 2014 and October 18, 2017 were downloaded from the laboratory information system (LIS) (Cerner Millenium, Kansas City, MO), following institutional review board (IRB) review and approval. The IRB did not require patient consent for this research. Protected health information (PHI) associated with laboratory results were stored on an encrypted and password protected departmental computer drive; only the investigators approved by the IRB were allowed to access this information. Irrelevant PHI was removed from the dataset at the earliest possible time, but due to the sensitive nature of HIV/AIDS testing and the fact that multiple non-PHI datapoints could be theoretically used to identify an individual, the dataset has not been made publicly available. Fourth-generation testing during this time period was performed exclusively on the Architect immunoassay platform (Abbott Laboratories, Lake Bluff, IL), which detects p24 antigen and antibodies to HIV-1 and HIV-2. Supplementary testing after November 1, 2016 was performed using the Geenius assay (Bio-Rad Laboratories, Hercules, CA); prior to this date the Multispot assay (Bio-Rad Laboratories, Hercules, CA) was used. Viral load testing was performed on plasma using the Roche CAPTAQ HIV-1 RNA assay (Roche Diagnostics, Basel, Switzerland).

Fourth-generation S/CO ratio data was obtained from backup disks of raw Architect data housed in our laboratory. Of note, data from the months of 3-6/2014, 10/2016, and 3/2017 were not available for analysis. All fourth generation tests were performed in duplicate per manufacturer instructions; to achieve the greatest sensitivity in our calculations, the higher of these two values was chosen for analysis, although analysis with averaged values yielded nearly identical results. Per manufacturer instructions when the initial testing was positive and repeat testing was negative the overall result was interpreted as negative. HIV testing data from each assay and source was collated for each patient encounter in Excel (Microsoft Corporation, Redmond, WA). Turn-around-time for patients with a “reactive” fourth-generation assay result but a negative supplemental result represents the time between the fourth-generation test result reporting and viral load test result reporting. To calculate turn-around-time, the date and time were extracted from BJH’s electronic health record viewer (Clinical Desktop) and then subtracted in Excel. Graphs and statistical analyses were performed in Prism (GraphPad Software Corporation, La Jolla, CA). All diagnostic testing performed during this study was compliant with respect to the College of American Pathologists (CAP) guidelines and criteria.

## Results

**Fig 1:**
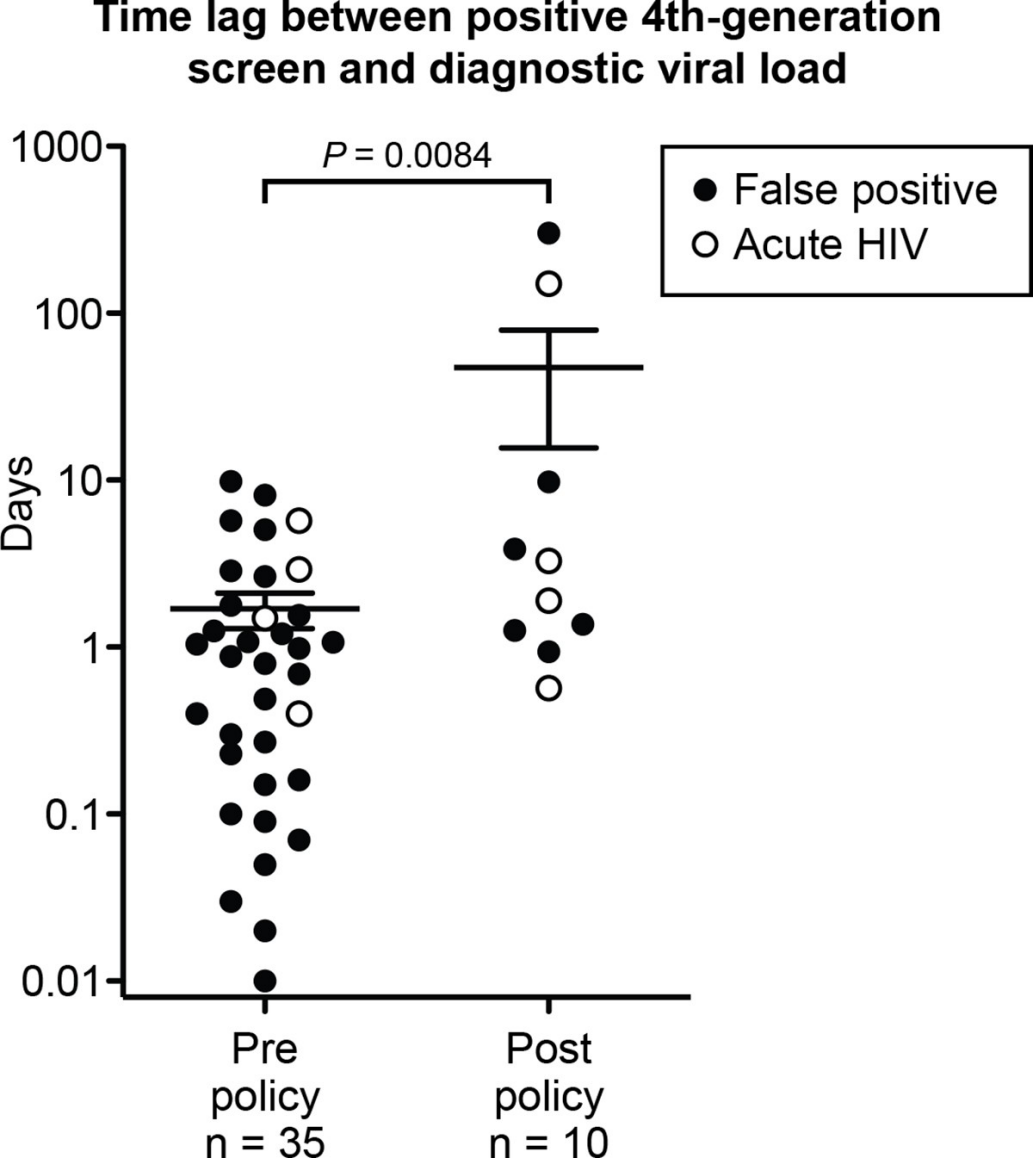
Requiring a dedicated specimen for molecular testing delays diagnosis for patients who have discrepant HIV screening and supplemental test results. Forty-five cases were identified in which the fourth-generation HIV screening assay was reactive but the supplemental assay was negative. All cases had subsequent viral load testing. The time between receipt of specimen for the fourth-generation test and receipt of specimen for viral load testing is shown, with testing that occurred prior (left) or after (right) the implementation of a dedicated-molecular-testing specimen policy. Specimens that were ultimately deemed to be false-positive by fourth-generation testing are shown in black, while those that were deemed to be acute HIV-positive are shown in white. Error bars show the mean with standard error of the mean. Statistical analysis performed using the Mann-Whitney test.

Between January 1, 2014 and October 18, 2017 we identified 45 patient encounters that resulted in a reactive fourth-generation screen but were negative by supplemental assay. All 45 of these patients were followed-up with diagnostic viral load testing, and 8 were subsequently found to be HIV-positive (17.7%). We observed a significant delay in the time-to-viral-load testing for encounters that occurred after the implementation of the dedicated-molecular-testing specimen policy on August 1, 2016 (Fig 1). Prior to policy implementation, the average time between fourth generation test and receipt of specimen for viral load testing was 1.7 days (median of 0.88 days), which increased to 47.5 days (median of 2.6 days) after implementation of the policy (p = 0.0084, Mann-Whitney test). This large increase was heavily influenced by the loss of two individuals to follow-up for >100 days (one of whom was unknowingly HIV-positive). Notably, no patients were lost to follow-up for >100 days prior to policy implementation, despite a >3-fold sample size for this group.

**Fig 2:**
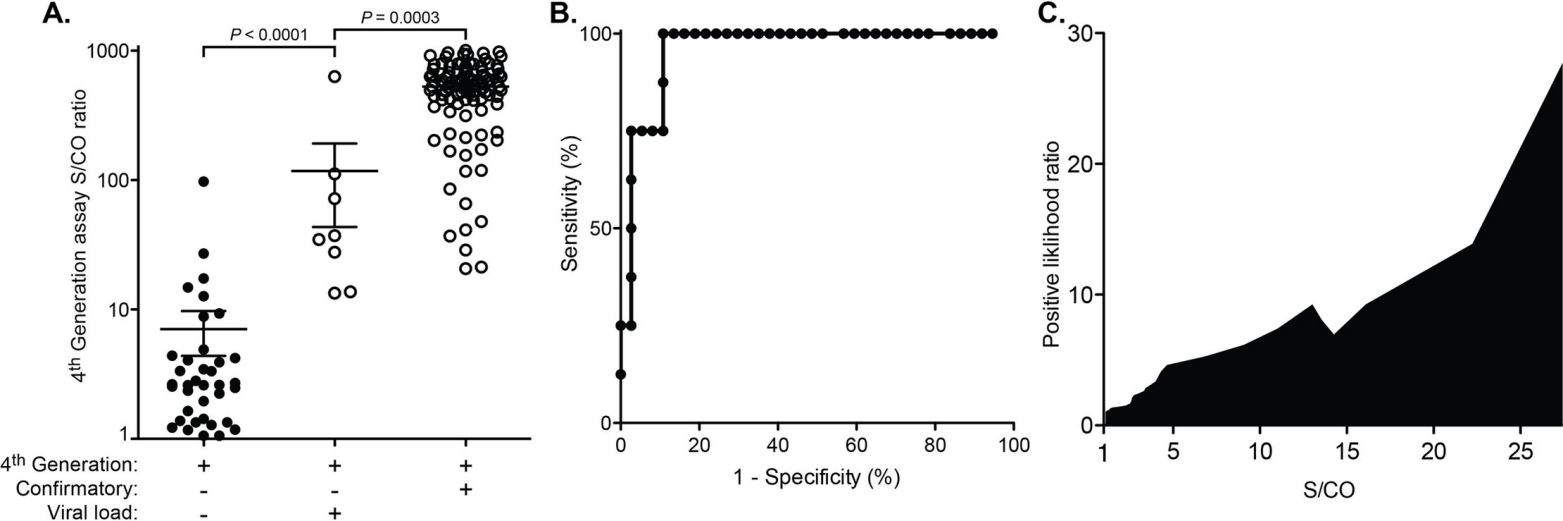
Fourth-generation assay signal-to-cutoff (S/CO) ratio results distinguish acute HIV-positive from false-positive results. (A) S/CO ratio values for false-positive (n = 37), acute-HIV infection (n = 8), and chronic/sub-acute HIV infection (n = 101) are shown. Error bars show the mean and standard error of the mean. Statistical analysis was performed using the Mann-Whitney test. A cutoff value of 13 has optimal performance characteristics, as determined by (B) a receiver operating characteristic (ROC) curve analysis which was also used to derive (C) a positive likelihood ratio per S/CO ratio value.

Given these results, we sought a method for presumptively distinguishing HIV-positive patients (*i*.*e*. those in the acute-phase of HIV infection) from HIV-negative patients (*i*.*e*. those with a false-positive fourth-generation assay result) when specimens were reactive by the fourth-generation assay but negative by the supplemental assay. The fourth-generation test performed on the Abbott Architect reports a specimen as “reactive” or “non-reactive.” However, this determination is based upon the quantitative signal-to-cutoff (S/CO) ratio, with a value >1 being the criterion that results in a sample being deemed “reactive.” When stratified by HIV status (as determined by molecular testing), S/CO values were significantly higher in specimens from the HIV-positive group (p<0.0001, Mann-Whitney test, Fig 2A). Receiver operating characteristic (ROC) curve analysis showed that the strength of S/CO positivity can distinguish true positive from false positive results with a high degree of confidence: using a S/CO ratio of 13 as a cutoff, this method has a sensitivity of 100% and a specificity of 89% (Fig 2B). The positive likelihood ratio also correlates well with the S/CO ratio value (Fig 2C). Of note, the S/CO ratio values observed for specimens that tested positive by the supplemental test (*i*.*e*. from patients in the chronic or sub-acute phase of HIV infection) were significantly higher than those that were reactive by the fourth-generation assay but negative by supplemental assay (Fig 2A).

## Discussion

Maintaining specimen integrity is a central tenet of molecular testing. However, we show here that requiring a dedicated specimen for molecular diagnosis of acute HIV infection delays time-to-diagnosis and increases loss-to-follow-up at a large academic medical center. Given that molecular testing is only typically used for diagnostic purposes when acute HIV infection is suspected, this finding has significant public health implications, as the risk of HIV transmission is greatest during the acute phase of infection when viral loads are highest [1, 2]. Because most HIV testing occurs in the outpatient and acute-care setting, we suspect that most encounters for patients receiving HIV screening are completed before test results are returned to the provider. Therefore, requiring a second specimen often necessitates a second encounter, which we hypothesize is a major contributor to delays in time-to-diagnosis and loss-to-follow-up. While splitting a specimen into multiple aliquots (or obtaining multiple specimens) prior to initiating the HIV testing algorithm could circumvent this issue, HIV screening is often high volume and a separate specimen is only required in rare circumstances, which renders this work-around cost-prohibitive for many institutions.

**Fig 3:**
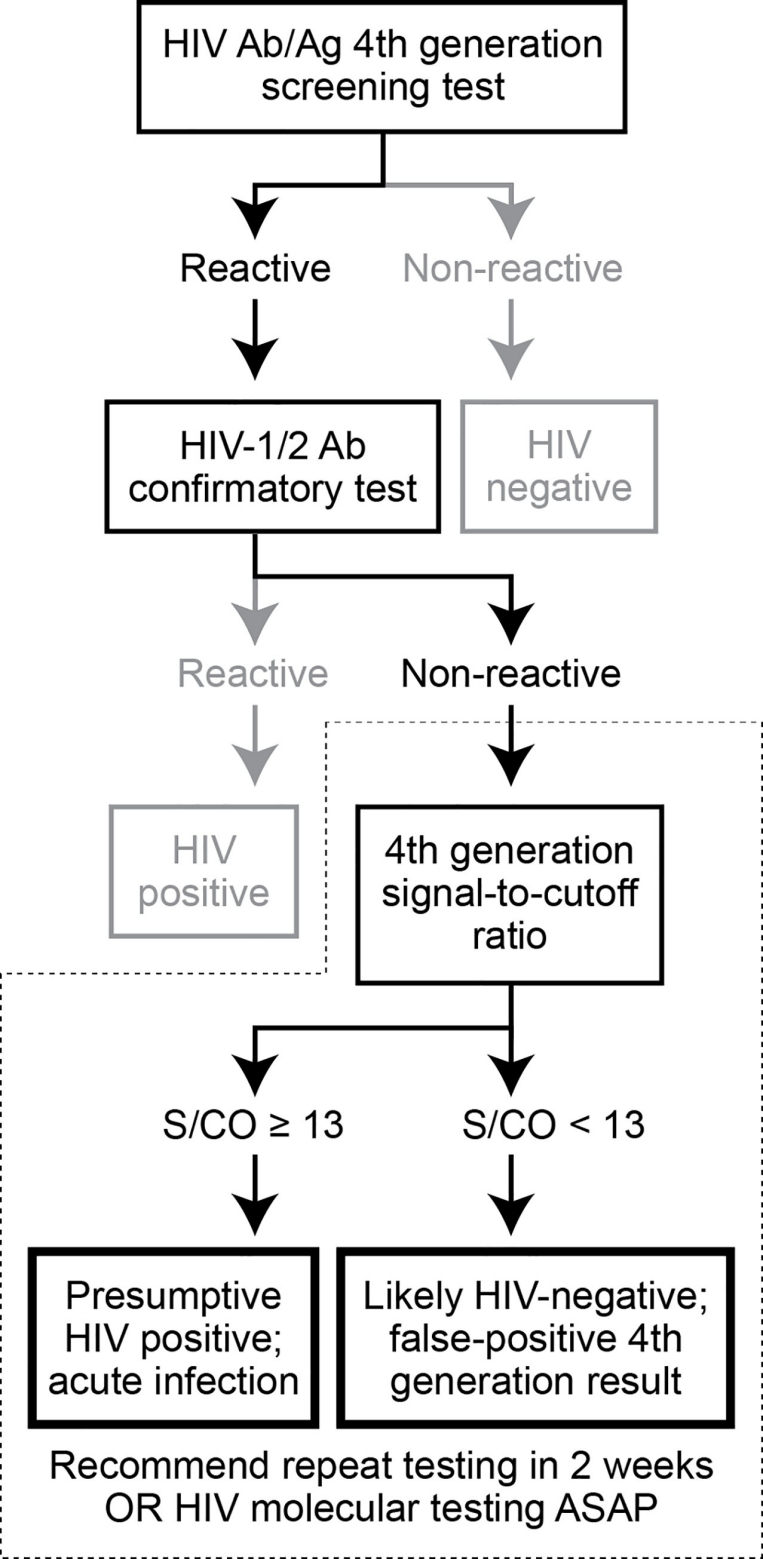
Proposed algorithm for evaluation of discrepant HIV testing results when molecular testing is not readily available. The standard HIV testing algorithm is shown, with discrepant results shown in dark black. The additional algorithm proposed herein is surrounded by a dashed line.

Given the importance of establishing a diagnosis of acute HIV irrespective of a specimen’s suitability for molecular testing, we propose an algorithm for predicting the likelihood of a true positive from specimens that are reactive by the fourth-generation test performed on the Abbott Architect but negative by supplemental testing and for which molecular testing cannot be immediately performed (Fig 3). This builds upon the existing HIV testing algorithm to stratify patients as “presumptive HIV positive” versus “likely false-positive fourth-generation screening test” based upon the S/CO ratio generated by the fourth-generation test. Multiple studies, including ours, have demonstrated high sensitivity and specificity when a S/CO ratio of >10–15 is useful to predict true HIV positivity [5–9]. Our study is unique in that we examined this cutoff in the specific patient population in which follow-up testing is challenging, those with positive fourth generation tests and negative supplemental tests. Although follow-up molecular testing is necessary in all patients with this testing pattern, identification of “presumptively positive” patients may be useful in allowing physicians to make early clinical decisions while awaiting confirmatory molecular testing. Presumptively positive patients may also warrant more aggressive follow-up measures and different forms of patient counseling. Interestingly, a very similar approach has recently been endorsed by the US CDC for hepatitis C virus (HCV) testing in settings where molecular confirmation is not readily available [10]. In these circumstances, platform-specific S/CO ratio cutoff values are provided–a practice that could also be extended to HIV screening platforms in the future. Data from our study would support this practice, though given the relatively small number of patients included in our study, a larger, multi-center, prospective study may be needed to more accurately define an optimal cutoff, and determine how this information is used in practice.

We believe that the algorithm addition proposed herein may be particularly helpful in settings such as smaller hospitals, clinics, and resource-limited settings where HIV testing is performed but molecular testing is not readily available; and in the emergency room setting where patients are often discharged before a second specimen for dedicated molecular testing can be obtained. As with all laboratory testing, the prevalence of HIV in the test population should be considered when using this algorithm. Additionally, this algorithm addition should not be used as an alternative to the standard fourth-generation HIV diagnostic algorithm, but rather as a tool that can help guide subsequent patient care and manage public health resources. By targeting aggressive measures for follow-up molecular testing to individuals who are “presumptive HIV positive,” we believe that implementation of this algorithm has the potential to decrease time-to-diagnosis for individuals with acute HIV infection and subsequently reduce HIV transmission.

In conclusion, requiring a dedicated molecular-testing specimen for the purposes of HIV diagnosis increases time-to-diagnosis and loss-to-follow-up. For patients in which acute HIV is possible (*i*.*e*. a negative supplemental assay), the S/CO ratio of the fourth-generation may be used to identify individuals who are “presumptive HIV positive.” Although not a substitute for eventual confirmatory molecular testing, this algorithm may be useful to clinicians in circumstances where a second specimen cannot be readily obtained and when molecular results are not immediately available.
